# Guaraná (*Paullinia cupana* Kunth) effects on LDL oxidation in elderly people: an *in vitro* and *in vivo* study

**DOI:** 10.1186/1476-511X-12-12

**Published:** 2013-02-08

**Authors:** Rafael de Lima Portella, Rômulo Pillon Barcelos, Edovando José Flores da Rosa, Euler Esteves Ribeiro, Ivana Beatrice Mânica da Cruz, Leila Suleiman, Felix Alexandre Antunes Soares

**Affiliations:** 1 Departamento de Química, Centro de Ciências Naturais e Exatas, Universidade Federal de Santa Maria, Campus UFSM, Santa Maria, RS, Brazil; 2 Universidade Aberta da Terceira Idade, Universidade do Estado do Amazonas, Amazonas, Brazil; 3 Departamento de Morfologia, Centro de Ciências da Saúde, Universidade Federal de Santa Maria, Campus UFSM, Santa Maria, RS, Brazil

**Keywords:** Lipoperoxidation, Humans, Antioxidant, Plant extract

## Abstract

**Background:**

Previous experimental investigations have suggested that guaraná (*Paullinia cupana* Kunth, supplied by EMBRAPA Oriental) consumption is associated with a lower prevalence of cardiovascular metabolic diseases and has positive effects on lipid metabolism, mainly related to low density lipoprotein (LDL) levels. As LDL oxidation is an important initial event in the development of atherosclerosis, we performed *in vitro* and *in vivo* studies to observe the potential effects of guaraná on LDL and serum oxidation.

**Methods:**

The *in vivo* protocol was performed using blood samples from 42 healthy elderly subjects who habitually ingested guaraná (GI) or never ingested guaraná (NG). The formation of conjugated dienes (CDs) was analyzed from serum samples. The *in vitro* protocols were performed using LDL obtained from 3 healthy, non-fasted, normolipidemic voluntary donors who did not habitually ingest guaraná in their diets. The LDL samples were exposed to 5 different guaraná concentrations (0.05, 0.1, 0.5, 1, and 5 μg/mL).

**Results:**

GI subjects demonstrated lower LDL oxidation than did NG subjects (reduction of 27%, p < 0.0014), independent of other variables. In the GI group the total polyphenols was positively associated with LDL levels. Also, guaraná demonstrated a high antioxidant activity *in vitro*, mainly at concentrations of 1 and 5 μg/mL, demonstrated by suppression of CDs and TBARS productions, tryptophan destruction and high TRAP activity.

**Conclusions:**

Guaraná, similar to other foods rich in caffeine and catechins such as green tea, has some effect on LDL oxidation that could partially explain the protective effects of this food in cardiometabolic diseases.

## Background

Foods rich in catechins and caffeine, such as tea and coffee, are the most popular beverages in the world, and have been consumed for thousands of years because of their alluring flavors and health benefits. Several epidemiological and experimental investigations have described an inverse association between tea consumption and cardiovascular diseases [1]. A recent study described an association between coffee and tea consumption and a low morbidity and mortality risk from stroke, coronary heart disease (CHD), and all causes of mortality in 37,514 subjects followed for 13 years [2]. A possible causal factor associated with tea and coffee consumption is the role of bioactive compounds present in these foods in metabolic pathways related to body weight loss, and a consequent reduction of the overall risk for developing metabolic syndrome [3].

However, it is important to analyze whether other foods rich in these compounds show biological properties similar to those of the guaraná used in energy drinks (*Paullinia cupana* H.B.K., Sapindaceae) [4]. Guaraná is a rainforest vine that was domesticated in the Amazon for its caffeine-rich fruits. During the last two decades, guaraná has emerged as a key ingredient in various ‘sports’ and energy drinks [5]. Energy drinks have increased in popularity with adolescents and young adults. Caffeine, the most physiologically active ingredient in energy drinks, is generally considered safe by the US Food and Drug Administration (FDA), although adverse effects can occur at varying amounts. Guaraná, which contains caffeine in addition to small amounts of theobromine, theophylline, and tannins, is also recognized as safe by the FDA [6]. Because the consumption of guaraná is growing in many countries, studies on its functional properties are needed.

Previous experimental investigations have suggested that guaraná has positive effects on lipid metabolism [7], in body weight loss [8], and increases basal energy expenditure [9]. Furthermore, studies suggested that guaraná exhibits a cardioprotective effect by inhibiting platelet aggregation [10]. These all positive effects contributed to reduce the risk factors for cardiovascular diseases. However, in contrast to green tea and coffee, on which many epidemiological studies have been performed, investigations involving guaraná consumption are difficult to perform because guaraná originates in a specific Brazilian Amazonian region (Maués-AM) [5]. Besides, there is no information whether guaraná consumption might increase the resistance to low density lipoprotein (LDL) oxidation.

For this reason, a controlled study was recently performed to analyze the association between habitual guaraná consumption and the prevalence of metabolic disease (obesity, hypertension, type 2 diabetes, and dyslipidemia) in an elderly population living in the Amazon’s Riverine region (Maués-AM). The study observed a lower prevalence of hypertension, obesity, and metabolic syndrome in the subjects which self-reported habitual guaraná consumption (GI) than in subjects who reported never ingesting guaraná (NG). Additionally an association was found between habitual guaraná consumption and lower cholesterol (total and LDL) and advanced oxidative protein product (AOPP) levels [11]. The potential effect of guaraná on LDL levels as well as oxidative biomarkers (AOPP) could provide a possible causal explanation for the lower prevalence of some cardiovascular metabolic diseases observed in Maués’s study.

These results lead us to verify whether guaraná may have a possible antiatherogenic activity. Lipid peroxidation induced by free radicals has been implicated in the pathogenesis of various diseases. Numerous *in vitro* and animal studies have shown that oxidative modification of LDL is an important initial event for the development of atherosclerosis [12]. Moreover, it is known that age is one of the major risk factor for atherosclerotic vascular disease [13]. Plasma cholesterol, triglyceride, LDL, polyunsaturated fatty acid (PUFA), total fatty acid and malondialdehyde levels, were found to increase in aged humans compared with young groups [14]. In addition, VLDL + LDL oxidizability increased and total thiol content levels in plasma decreased in aged humans and rats compared with young groups [14].

The experimental studies described earlier and the established inverse relationship between the consumption of fruit and vegetables and cardiovascular diseases have led to a number of new studies on patients and populations that, for the most part, seem to reinforce the central role of antioxidants as protective nutrients [13]. Considering these facts, we performed an *in vivo* study to investigate the potential effects of guaraná in elderly people on serum oxidation and an *in vitro* study in order to investigate the antioxidant effects of guaraná on the LDL and serum oxidation.

## Results

### Baseline characteristics of subjects

The baseline characteristics of GI and NG elderly subjects included in the *in vivo* serum oxidation assay are presented in Table 1. In general, the two elderly groups were similar in BMI, blood pressure, glucose levels, and certain lipid parameters. However, the levels of total cholesterol were higher in the NG subjects than in the GI subjects. Additionally, the oxidative biomarker parameters such as TBARS, total polyphenols, and protein carbonylation were similar for the two groups.

**Table 1:** Parameters from people who habitually ingest guaraná (GI) and those who never ingest guaraná (NG)

**Variables**	**GI**	**NG**	***p***
	**Means ± SD**	**Means ± SD**	
Age (years)	73.6 ± 6.5	75.2 ± 9.4	0.56
BMI (Kg/m^2^)	27.4 ± 3.3	27.0 ± 4.2	0.58
Waist circumference (cm)	87.6 ± 11.1	82.5 ± 10.9	0.14
SBP (mmHg)	126.1 ± 15.9	130.0 ± 15.8	0.44
DBP (mmHg)	73.51 ± 8.3	76.51 ± 4.9	0.19
Glucose (mg/dL)	103.7 ± 12.8	108.6 ± 14.1	0.21
Cholesterol total (mg/dL)	189.7 ± 30.8	230.2 ± 61.6	0.009
Triglycerides (mg/dL)	128.7 ± 47.7	150.9 ± 51.7	0.57
LDL (mg/dL)	132.5 ± 45.8	161.1 ± 52.6	0.08
HDL (mg/dL)	42.6 ± 24.0	42.2 ± 19.1	0.96
Uric acid (mg/dL)	4.2 ± 1.9	5.4 ± 2.9	0.16
TBARS	21.7 ± 7.9	19.9 ± 7.0	0.44
Protein carbonilation	0.17 ± 0.09	0.14 ± 0.07	0.25
Total polyphenols	2.7 ± 0.8	2.7 ± 0.5	0.98

SD = standard deviation; BMI = Body mass index (Kg/m^2^); SBP = systolic blood pressure; DBP = diastolic blood pressure. Comparison between GI and NG elderly subjects performed by *t-student* test. *p* value = statistical significance.

### Conjugated diene levels in Maués’s population

**Figure 1:**
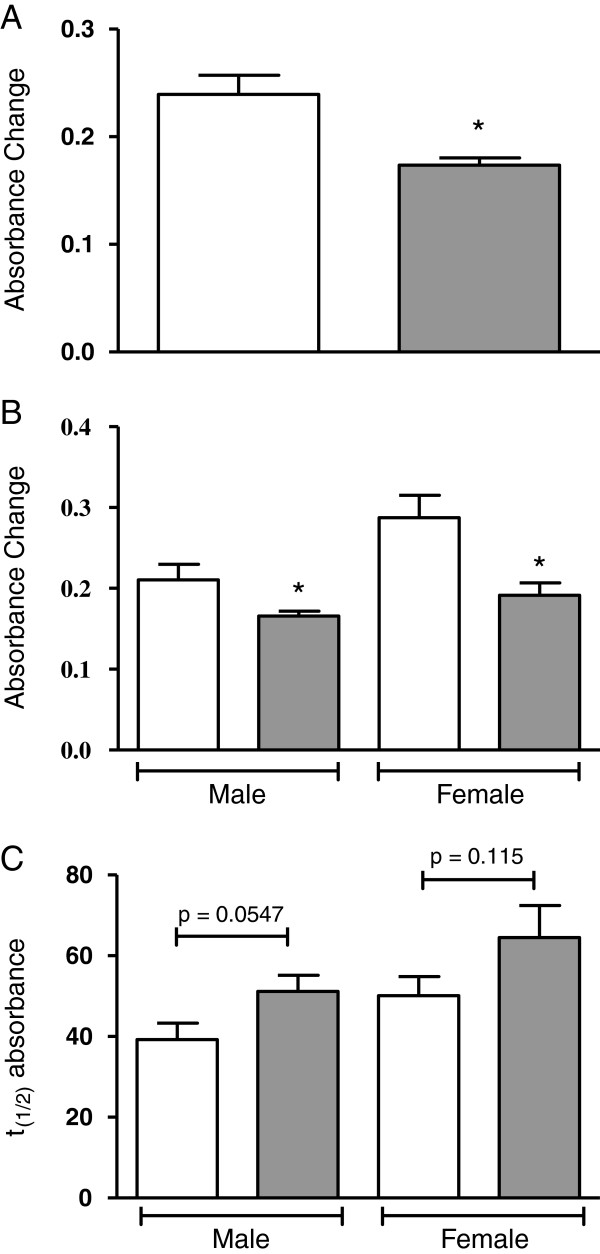
**Maximum conjugated dienes production in serum from Maués inhabitants.** Serum (diluted 100-fold) in PBS 10 mM (pH7.4) was incubated at 37°C for 180 min in the presence of 30 μM CuSO_4_. Conjugated diene formation was measured by determining the absorbance at 245 nm every 20 min. In panel **A**, maximum CD production in serum of GI and NG subjects. In panel **B**, gender effect on maximum CD production between GI and NG groups. NG (white bars) and GI (gray bars). In panel **C**, the t_(1/2)_ indicates the time required to achieve 50% CD production. * p < 0.05 compared to white bar.

The serum samples from elderly GI and NG subjects were evaluated for maximum CD production. The diene levels were significantly different between the two groups analyzed here. The GI group showed lower diene formation (reduction of 27%, p < 0.0014) than the NG group (Figure 1A). The potential gender effect on diene formation in GI and NG groups was also analyzed. Figure 1B shows that both men (reduction of 21%, p < 0.0168) and women (reduction of 33%, p < 0.0311) who drank guaraná showed a significant decrease in maximum CD production compared to their respective gender subjects that did not drink guaraná. Figure 1C shows the time required to achieve 50% CD production during CuSO_4_-induced serum oxidation. This result shows no significant difference between NG and GI groups. Pearson correlation tests were performed and there are no significant correlations between maximum conjugated dienes and either total cholesterol (r = 0.188, p = 0.23) or LDL levels (r = 0.137, p = 0.39). However, we found a significant correlation between total polyphenols and LDL levels in the GI group (r = 0.643, p = 0.007) but not in the NG group (r = 0.197, p = 0.563).

### *In vitro* guaraná effect on LDL oxidation

#### Conjugated diene (CD) production

**Figure 2:**
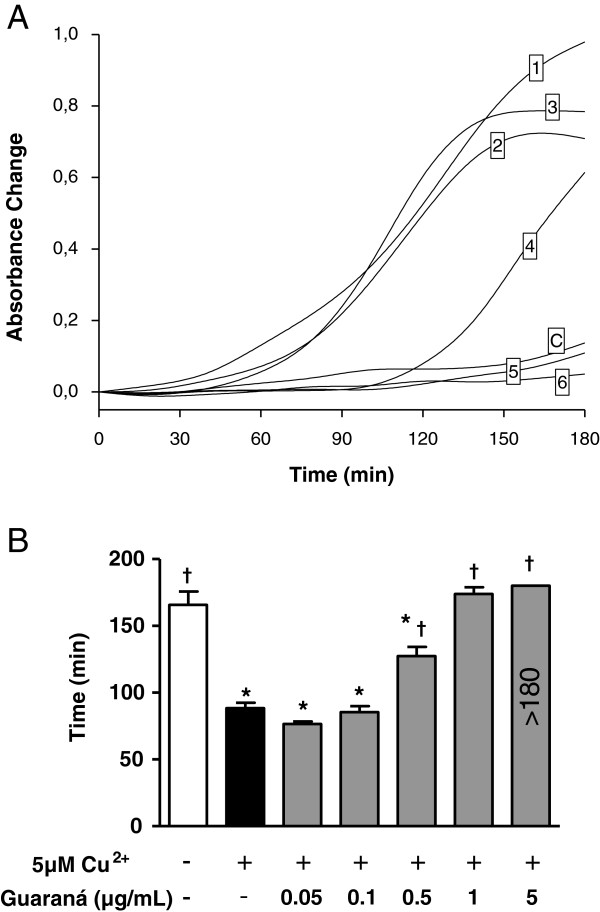
**Effects of guaraná on LDL oxidation.** LDL (50 μg protein/mL) was incubated in PBS 10 mM (pH 7.4) at 37°C in the presence of CuSO_4_ 5 μM. Conjugated diene formation was measured by determining the absorbance at 234 nm every 20 min. In panel **A**, incubation medium did not contain guaraná (1) or contained 0.05 μg/mL (2), 0.1 μg/mL (3), 0.5 μg/mL (4), 1 μg/mL (5) or 5 μg/mL (6) of guaraná and control without CuSO_4_ and guaraná (C). In panel **B**, the value of the lag phase determined graphically by the intercept of the tangents to the slow and fast increase of the diene absorption. Experiments were repeated six times, showing similar results. * p < 0.05 compared to group without CuSO_4_ and guaraná. † p < 0.05 compared to group with CuSO_4_ and without guaraná.

Isolated LDL samples incubated with different concentrations of guaraná showed a concentration-dependent increase in the lag phase of LDL oxidation (r^2^ = 0.88, p < 0.001). Guaraná concentrations in the range of 0.5 – 5 μg/mL significantly inhibited CuSO_4_-induced LDL oxidation, increasing the lag phase (Figure 2A). At concentrations of 1 and 5 μg/mL, guaraná totally prevented LDL oxidation during an assay time of 180 min (Figure 2B). However, guaraná had no effect on the maximum rate of oxidation (data not shown).

#### TBARS production

**Figure 3:**
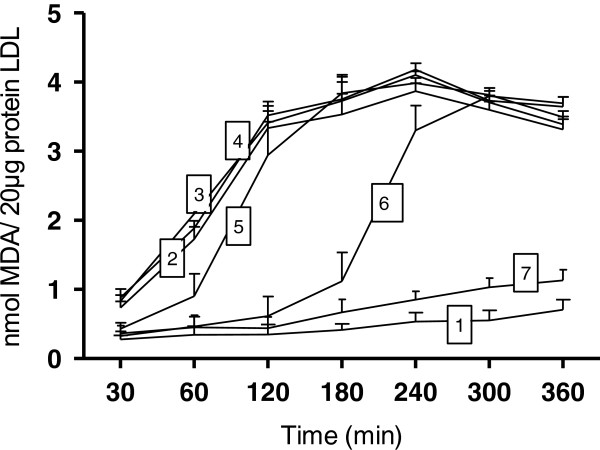
**Effects of guaraná on thiobarbituric acid reactive substances (TBARS) production during CuSO**_**4**_**-induced LDL oxidation.** LDL (50 μg protein/mL) was incubated in PBS 10 mM (pH 7.4) at 37°C in the presence of CuSO_4_ 5 μM. Incubation medium did not contain guaraná and CuSO_4_ (1) or contained CuSO_4_ (2), 0.01 μg/mL (3), 0.05 μg/mL (4), 0.1 μg/mL (5), 1 μg/mL (6) or 5 μg/mL of guaraná. Experiments were repeated three times.

LDL oxidation was also evaluated by TBARS formation. Figure 3 shows a significant effect of guaraná in preventing TBARS formation induced by CuSO_4_. The protective effect of guaraná was similar to that demonstrated in the CD experiments. Guaraná at 1 μg/mL showed a significant effect during an assay time of 60 – 180 minutes. At a concentration of 5 μg/mL, guaraná prevented TBARS formation during an assay time of 60 – 360 min.

#### LDL tryptophan fluorescence

**Figure 4:**
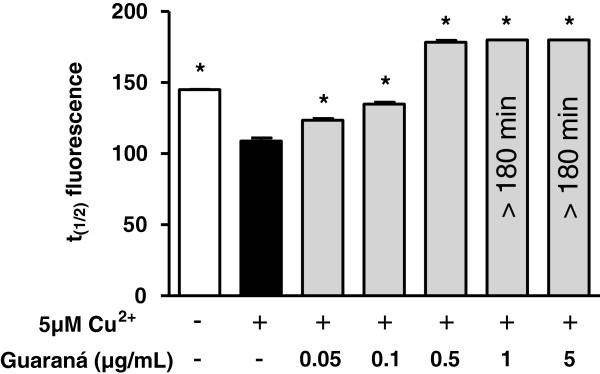
**Effect of guaraná on Trp destruction in CuSO**_**4**_**-induced LDL oxidation.** LDL (50 μg protein/mL) was incubated in PBS with 5 μM CuSO_4_ with different guaraná concentrations. Fluorescence (Ex/Em = 282/331 nm) was measured at intervals of 20 min at 37°C. The t_(1/2)_ means the time required for reaching half Trp fluorescence. Experiments were repeated three times, showing similar results. * p < 0.05 compared to control with copper and without guaraná (black bar).

Figure 4 shows the time required to achieve 50% Trp fluorescence (t_1/2_) during CuSO_4_-induced LDL oxidation. When compared to the control group, the presence of guaraná at all concentrations tested significantly increased the t_1/2_ of Trp. Guaraná at 1 and 5 μg/mL showed a total protection during 180 minutes. This result was similar that for the conjugated dienes and TBARS formation.

### CD production in serum

**Figure 5:**
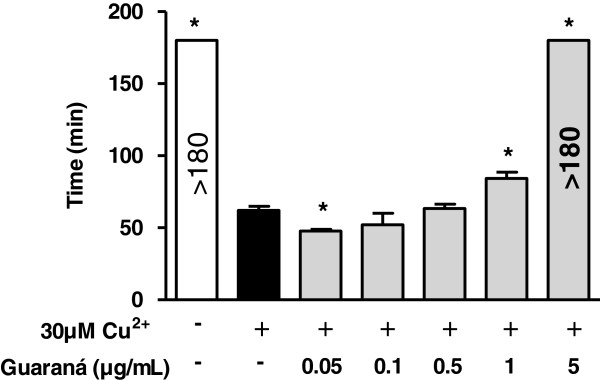
**Effects of guaraná concentrations on phase lag of conjugated dienes formation during serum oxidation.** Serum (diluted 100-fold) in PBS 10 mM (pH7.4) was incubated at 37°C for 180 min in the presence of 30 μM CuSO_4_. Conjugated diene formation was measured by determining the absorbance at 245 nm every 20 min. Experiments were repeated three times, showing similar results. * p < 0.05 compared to control with copper and without guaraná (black bar).

Serum oxidation was determined by CD formation at 245 nm. No oxidation occurred in serum when the medium did not contain CuSO_4_. Figure 5 shows that guaraná at concentrations of 1 and 5 μg/mL was able to cause a significant increase in the lag phase of oxidation. At the highest concentration, guaraná totally inhibited the serum oxidation during the assay time. Similar to its effect on LDL oxidation, guaraná demonstrated no effect on the maximum oxidation rate in serum oxidation (data not shown).

### Total radical-trapping antioxidant potential of guaraná

**Figure 6:**
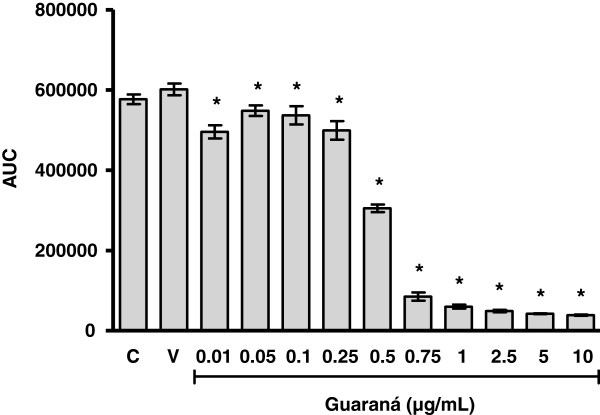
***In vitro *effect of guaraná on total radical-trapping antioxidant potential (TRAP).** The reaction mixture contained 10 mM AAPH, 35 μM luminol and guaraná at different concentrations dissolved in 0.1 M glycine buffer (pH 8.6). The luminescence was measured every 5 minutes during 5 hours. The area under curve (AUC) was evaluated. (V) Vehicle and (C) Control. The bars represent mean ± S.D. of five different experiments. * p < 0.001 compared to control and vehicle group. There is no difference between vehicle and control group.

Figure 6 shows the antioxidant potential of guaraná when AAPH was used as pro-oxidant. This method is based on the use of a water-soluble azo compound, AAPH [2,20-azo-bis(2-amidinopropane)-dihydrochloride], as a reliable and quantifiable source of alkyl peroxyl radicals. The thermal decomposition of these compounds in the presence of luminol produces luminescence, which is quenched by the addition of peroxyl radical scavengers [15]. Guaraná concentrations ranging from 0.01 to 10 μg/mL were used in this experiment. We observed that all concentrations tested were able to reduce the AAPH-induced luminol oxidation, and that guaraná concentrations of 0.5 – 10 μg/mL demonstrated very strong inhibition.

## Discussion

To our knowledge, this is the first study conducted to investigate the potential association between guaraná effect on LDL and serum oxidation. The results showed that guaraná ingestion (GI) resulted in lower maximum conjugated diene production than that found in an elderly NG group (Figure 1). Furthermore, *in vitro* assays showed that guaraná increased the lag phase in the oxidation of LDL and serum *in vitro* (Figures 2 and 5), and also prevented TBARS production (Figure 3) and Trp destruction (Figure 4) in LDL oxidation. Moreover, we observed that guaraná extract demonstrates a peroxyl radical scavenger activity (Figure 6).

The effects of guaraná on LDL and serum oxidation are probably associated with some bioactive compounds (catechins and xantines) that are similar to those found in other foods, such as green tea [4]. Tea polyphenols are well studied and there are numerous studies consistently describing these compounds as having antioxidative, antithrombogenic, antiinflammatory, hypotensive, hypocholesterolemic, antihypertensive, and antiobesogenic effects [16]. The potential beneficial effects of daily guaraná consumption described by Krewer et al [11] include a lower prevalence of hypertension, obesity, and metabolic syndrome, and lower cholesterol (total and LDL) and AOPP levels in GI subjects. These effects are similar to those described in epidemiological studies involving green tea [17]. We believe that the results depicted here concerning the LDL oxidation could contribute in the elucidation of potential causal factors related to these associations.

First, it is important to consider the bioactive effects of guaraná related to the main chemical compounds. The antioxidant effects of guaraná extract might be due to methylxanthines, such as caffeine, theobromine, and theophylline, and also to tannins, saponins, catechins, epicatechins, proanthocyanidols, as well as trace concentrations of many other compounds [18]. Effect of caffeine on LDL resistance to oxidative modification has been excluded by several *in vitro*[19] and *ex vivo*[20] studies. Indeed, we tested the TRAP activity of caffeine and theobromine and we observed no effect for these two compounds (data not shown). On the other hand, caffeine has been linked to increased thermogenesis and decreased body weight in some clinical studies [21]. These effects of caffeine may contribute to prevent the other risk factors related to atherosclerotic vascular disease such as waist circumference and blood pressure [11].

It has been shown that guaraná inhibits the lipid peroxidation process, an effect apparently associated with the high tannin content of the seeds, which reach concentrations between 16% and 31% [22,23]. Fresh tea leaf is unusually rich in the flavanol group of polyphenols known as catechins which may constitute up to 30% of the dry leaf weight and there is no tannic acid in tea [24]. Tannins are polyphenolic compounds having molecular masses between 500 and 3000 Da and a sufficiently large number of phenolic groups [25]. Yamaguti-Sasaki et al. [22] identified some procyanidins (condensed tannins) as epicatechin-(4β → 8)-epicatechin (procyanidin B2), catechin-(4α → 8)-catechin (procyanidin B3), and catechin-(4α → 8)-epicatechin (procyanidin B4). It has been demonstrated that green tea catechins demonstrate antioxidant activity by scavenging free radicals and chelating redox active transition-metal ions [26]. Catechins have many phenolic hydroxyl groups in their structures and have been shown to inhibit oxidative modification of LDL when added before initiation of oxidation [27]. However, the mechanisms by which these flavonoids inhibit LDL oxidation have not been clarified. Because of their amphipathic nature, flavonoids may act within the LDL particle in a manner similar to that of vitamin E, or may act in a manner comparable with that of ascorbic acid in the extraparticle environment of LDL. Considering our *in vivo* results (Figure 1) and the significant correlation between the total polyphenols and the LDL levels in the GI group, we may suppose that polyphenols from guaraná could incorporate into LDL, turning the serum from GI subjects less susceptible to oxidation *in vitro*.

Guaraná’s effect on lipid peroxidation *in vitro* is in agreement with Mattei et al [23], who showed that guaraná extract inhibited lipoperoxidation even at low concentrations (1.2 μg/mL). Besides their *in vitro* effects, catechins demonstrate additional effects *in vivo* by inhibiting redox active transcriptional factors, inhibiting pro-oxidant enzymes, and inducing antioxidant enzymes, which could explain their beneficial effects *in vivo*[26]. These effects make the potential use of guaraná extract even most promising to promote protection against atherogenesis.

In our *in vivo* study, the diene levels were significantly lower in GI than in NG subjects. It may indicate that guaraná intake is able to provide an additional antioxidant protection to serum and, mainly, to LDL. It was in agree with our *in vitro* data, which could increase the lag phase of serum and LDL oxidation, prevent TBARS production and tryptophan destruction (Figures 2, 3, 4 and 5). However, the time required to achieve 50% CD production was not significant different. We believe that it was not significant because of the low number of subjects. In the actual *in vivo* condition, the polyphenols and methylxanthines in plasma may work together to prevent LDL oxidation. These polyphenols may be more easily incorporated into LDL *in vivo* than *in vitro*. Although guaraná polyphenols may be metabolized quickly after entering the circulation, it is possible that these metabolites also exert preventive effects on LDL oxidation. Repeated exposure of LDL particles to guaraná polyphenols over a long period of time may enrich the LDL particles sufficiently to make them less susceptible to oxidative stress.

It is important to ponder some considerations associated with the methodological design of our *in vivo* protocol. Since the *in vivo* study was performed using a group of elderly subjects, and without controlling for the amounts of guaraná ingested or the ingestion of other foods rich in antioxidants or bioactive compounds, it is important to conduct additional controlled studies on the possible effect of guaraná ingestion on LDL oxidation levels to confirm the results described here.

## Conclusion

In summary, *in vivo* results showed that guaraná intake could reduce the diene levels in serum from elderly subject. This positive effect of guaraná could be confirmed by *in vitro* results which showed an increased resistance to LDL oxidation. It was due to high content of polyphenolic compounds, which may act to prevent atherogenesis through a combination of effects, including the other positive effects of guaraná on lipid metabolism [7], in body weight loss [8], and increases basal energy expenditure [9], besides the lower prevalence of hypertension, metabolic syndrome, and lower cholesterol and AOPP levels in GI subjects [11]. Considering these results, our study indicates that consumption of guaraná regularly or its possible inclusion in diet-based therapies could yield certain health benefits and potential defense against oxidative stress and metabolic disorders.

## Materials and methods

### *In vivo* assay: effect of habitual guaraná consumption on serum oxidation

The elderly patients were classified into two groups based on self-reported data: those who habitually ingested guaraná (at least 5 times a week; GI) and those who never ingested guaraná (NG). From a previous data and biological bank of the study performed by Krewer et al [11] that investigated elderly included in GI (n = 421) and NG groups (NG, n = 239) we select 42 samples from 22 males and 20 females (≥ 60 years of age, 23 GI and 19 NG) to perform a serum oxidation analysis. We selected subjects without previous life style as smoking habit and higher alcoholic beverage consumption and without morbidities that could to influence the analysis of serum oxidation as: type 2 diabetes mellitus, obesity, dyslipidemia, severe hypertension, metabolic syndrome, cardiovascular diseases, neoplasias and other metabolic diseases. The methodology used to determine biochemical parameters from elderly Riverine inhabitants who habitually ingest guaraná (GI) and those who never ingest guaraná (NG) are described in materials and methods from Krewer et al [11].

The Maués elderly population study was approved by the Ethical Committee of the Universidade do Estado do Amazonas, Brasil (nº 807/04). Since the vast majority of the elderly included in this study were illiterate, oral consent or fingerprint in Term was obtained to indicate their voluntary participation in the study after the researchers read the consent form to the patients.

Venous blood was drawn from these 42 previous selected elderly subjects into tubes containing no anticoagulant and centrifuged at 1000 g for 15 min and the serum was stored at −20°C until to be analyzed. Serum diluted 100-fold was incubated at 37°C in a medium containing 10 mM phosphate buffer (pH 7.4). The oxidation was initiated by the addition of CuSO_4_ (30 μM, final concentration) and conjugated dienes (CD) formation was monitored at 245 nm as previously described [28].

### Guaraná extract

Powdered *Paullinia cupana* Kunth seed produced and supplied by EMBRAPA Oriental (Agropecuary Research Brazillian Enterprise) located Western Amazon in Maués, Amazonas-Brazil was used in all experiments. The guaraná powder was conserved in dry conditions at ± 4°C, protected against light action until the extracts preparations.

We used a hydro-alcoholic extract of *Paullinia cupana* Kunth using alcohol and water (70:30) to 100 mL of extraction fluid prepared at a concentration of 300 mg/mL. The detailed description and determination of mainly bioactive compounds presents guaraná extract used in this study is presented in Bittencourt et al (Bittencourt LS, Machado DC, Machado MM, Santos GFF, Algarve TD, Marinowic DR, Ribeiro EE, Soares FAA, Athayde ML, Cruz IBM, unpublished data, 2011). Briefly, after 21 days of guaraná extraction the extract was centrifuged for 1000 g during 10 min and the supernatant was isolated and lyophilized. The mainly xanthines and catechins presented in guaraná extract were analyzed by chromatography and from this analysis was found caffeine = 12.240 mg/g, theobromine = 6.733 mg/g, total catechins = 4.336 mg/g, and condensed tannins = 22 mg/g.

The guaraná solution used in the study was prepared based on Santa Maria et al [29] protocol. The extract obtained and lyophilized was diluted in distillated water prepared at a concentration of 200 mg/mL. The mixture was infused for 7 min in boiling, and centrifuged (600 g, 15 min) and filtered. Five guaraná concentrations were tested here: 0.05, 0.1, 0.5, 1 and 5 μg/mL. In TRAP assay were used different guaraná concentrations (0.01-10 μg/mL).

### LDL isolation to *in vitro* protocol tests

To perform *in vitro* LDL-oxidation assays in the presence of guaraná, firstly the LDL was isolated from fresh human plasma by discontinuous density-gradient ultracentrifugation as described by Silva et al [30], with few modifications. Briefly, plasma of three healthy normolipidemic voluntary donors that did not ingest guaraná in their habitual diet collected with EDTA (1 mg/mL) was pooled and sucrose (final concentration, 0.5%) was added to prevent LDL aggregation. Five milliliters of EDTA-plasma adjusted to a density of 1.22 g/mL with solid KBr (0.326 g/mL) was layered on the bottom of a centrifuge tube. Then, 5 mL EDTA-containing sodium chloride solution (density 1.006 g/mL) was overlaid on the top of the plasma. Ultracentrifugation was run at 350,000 g for 2 h at 4°C, in a Himac CP80MX ultracentrifuge. LDL particles were collected by the aspiration of the yellow/orange band at the middle of the saline layer and dialyzed exhaustively overnight at 4°C with 10 mM phosphate buffer (pH 7.4). Protein concentration in LDL solution was determined by Lowry’s method [31]. The purity of LDL preparation was verified by agarose gel electrophoresis. Isolated LDL was stored at −20°C for no longer than 2 weeks.

### *In vitro* LDL oxidation analysis

#### Conjugated dienes and TBARS formation

LDL samples (50 μg protein/mL) were pre-incubated at 37°C in a medium containing 10 mM phosphate buffer (pH 7.4) and different guaraná concentrations (0.05 – 5 μg/mL). After 5 minutes, the oxidation was initiated by the addition of CuSO_4_ (5 μM, final concentration). The oxidation was monitored by measuring the increase in absorbance at 234 nm due to conjugated diene (CD) formation as previously described [32]. Aliquots were also removed at different time points for evaluating thiobarbituric acid reactive substances (TBARS) production as previously described [33].

#### Measurement of LDL- Tryptophan fluorescence

The fluorescence spectra of native LDL display a single band centered at approximately 332 nm, which is assigned to the tryptophan (Trp) residues in apolipoprotein B-100 (apoB-100) [34]. Loss of Trp fluorescence is a marker for oxidations at the protein core of LDL [34]. Trp fluorescence was measured in a solution of LDL (50 μg protein/mL) in PBS (10 mM) pH 7.4 at 37°C, using a Shimatzo espectrofluorometer (excitation at 282 nm and emission at 331 nm) [34]. The kinetics of LDL oxidation was followed by measuring the decrease of Trp-fluorescence, corresponding to the decomposition of this amino acid, after the addition of CuSO_4_ (5 μM, final concentration), in absence or presence of guaraná (0.05 – 5 μg/mL). The cuvettes had to be removed from the excitation light between the single measurements to avoid photooxidation of the Trp residues; fluorescence was measured every 20 min. Data are shown as the percent decrease of Trp fluorescence in each sample. The time required for reaching half Trp fluorescence (t_1/2_) was calculated.

### *In vitro* serum oxidation

Venous blood was drawn from nonfasted healthy normolipidemic voluntary donors into tubes containing no anticoagulant and centrifuged at 1000 g for 15 min. Serum diluted 100-fold was incubated at 37°C in a medium containing 10 mM phosphate buffer (pH 7.4) and different guaraná concentrations (0.05 – 5 μg/mL). The oxidation was initiated by the addition of CuSO_4_ (30 μM) and CD formation was monitored at 245 nm as previously described [28].

### Determination of lag phase and maximum oxidation rate

In the studies of CD formation, there are several parameters which can be obtained from diene vs. time profiles. The value of the lag phase is commonly determined graphically by the intercept of the tangents to the slow and fast increase of the diene absorption. Another parameter is the maximum oxidation rate, given by the peak of the first derivative, i.e. change of A_234_ as a function of time [32].

### Total radical-trapping antioxidant potential (TRAP)

TRAP was determined by measuring the chemiluminescence intensity of luminol induced by [2,20-azo-bis(2-amidinopropane)-dihydrochloride] (AAPH) thermolysis in a luminometer BioTek Synergy 2 [15]. The reaction mixture contained AAPH (10 mM) and luminol (35 μM) dissolved in 0.1 M glycine buffer (pH 8.6). Incubation of this mixture generates an almost constant light intensity at room temperature after stabilization. Guaraná was added in different concentrations to determine the TRAP activity. At this point, the luminescence intensity is practically abolished. In the course of time, with the loss of antioxidant capacity of guaraná, the luminescence intensity returns to the initial values. The area under curve (AUC) was evaluated for each guaraná concentration and compared to vehicle AUC [15].

### Statistical analysis

Statistical analysis was performed using the SPSS statistical package, version 17.0 (SPSS, Inc., IL). Data are expressed as means ± SD. Comparison between characteristics baselines and serum oxidation of GI and NG elderly subjects was performed by Student *t*-test. Multivariate logistic regression (Backward Wald method) and Pearson correlation tests were performed to observe possible intervenient factors. *In vitro* LDL-oxidation assays were statistically analyzed using a one-way analysis of variance (ANOVA), followed by Student-Newman-Keuls test when appropriate. In addition, linear regression was performed to identify a possible dose dependent effect. Values of p < 0.05 were considered significant.

## Abbreviations

AAPH: 2,2′-Azobis(2-methylpropionamidine) dihydrochloride; AOPP: Advanced oxidative protein product; apoB-100: Apolipoprotein B-100; AUC: Area under curve; CD: Conjugated diene; CHD: Coronary heart disease; FDA: Food and Drug Administration; GI: Guaraná intake; NG: No guaraná intake; LDL: Low density lipoprotein; HDL: High-density lipoprotein; MDA: Malondialdehyde; PUFA: Polyunsaturated fatty acid; TBARS: Thiobarbituric acid reactive substances; TRAP: Total radical-trapping antioxidant potential; Trp: Tryptophan; VLDL: Very low density lipoprotein

## Competing interests

The authors declare that they have no competing interests.

## Authors’ contributions

The authors’ responsibilities were as follows: RLP was involved in data collection, data analysis, data interpretation, literature search and manuscript preparation; RPB, EJFR, EER and LS were involved in data collection, data analysis and data interpretation; IBMC and F.A.A.S. were involved in the study design, data interpretation and review of the manuscript; all authors contributed to the revision of the manuscript and final approval of the manuscript.
